# Effect of Duration and Amplitude of Direct Current when Lidocaine Is Delivered by Iontophoresis

**DOI:** 10.3390/pharmaceutics3040923

**Published:** 2011-12-06

**Authors:** Susan A. Saliba, Courtney L. Teeter-Heyl, Patrick McKeon, Christopher D. Ingeroll, Ethan N. Saliba

**Affiliations:** 1 University of Virginia, PO Box 400407, Charlottesville, VA 22904, USA; 2 Ortho Rehab & Specialty Centers, 3808 Rose Point Cove, PO Box 241574, Little Rock, AR 72223,USA; E-Mail: clt9b@yahoo.com; 3 University of Kentucky, 900 South Limestone Street, Lexington, KY 40536, USA;E-Mail: McKeon@uky.edu; 4 Central Michigan University, 2217 Health Professions Building, Mt. Pleasant, MI 48859, USA;E-Mail: inger1c@cmich.edu; 5 University of Virginia, PO Box 400834, Charlottesville, VA 22904, USA; E-Mail: ens@virginia.edu

**Keywords:** percutaneous drug delivery, physical therapy, transdermal, electrical stimulation, electroporation

## Abstract

Dosage for the galvanic stimulation for iontophoresis varies. Clinicians manipulate the duration or the amplitude of the current, but it is not known which is more effective. We compared the anesthetic effect of lidocaine HCL (2%) by manipulating the current parameters on 21 healthy volunteers (age: 21.2 ± 4.2, height 170.7 ± 10.2 cm, mass 82.1 ± 19.2 kg). Three conditions were administered in a random order using a Phoresor II^®^ with 2 mL, 2% lidocaine HCL in an iontophoresis electrode. (1) HASD (40 mA_*_min): High amplitude (4 mA), short duration (10 min); (2) LALD (40 mA.min): Low amplitude (2 mA), long duration (20 min); (3) Sham condition (0 mA, 20 min). Semmes-Weinstein monofilament (SWM) scores were taken pre and post intervention to measure sensation changes. Two-way ANOVA with repeated measures was used to compare sensation. Both iontophoresis treatments: LALD (4.2 ± 0.32 mm) and HASD (4.2 ± 0.52 mm) significantly increased SWM scores, indicating an increase in anesthesia, compared to the sham condition (3.6 ± 0.06 mm) p < 0.05. Neither LALD nor HASD was more effective and there was no difference in anesthesia with the sham. Lidocaine delivered via iontophoresis reduces cutaneous sensation. However, there was no benefit in either a HASD or LALD treatment.

## 1. Introduction

Iontophoresis is a noninvasive method of electrically administering medications in their ionic form into the body through the use of a direct current [1,2]. Iontophoresis is sterile, noninvasive, is less painful than a local injection and avoids the first pass through the hepatic system. Furthermore, iontophoresis can be applied directly to an injured or painful site, resulting in a local effect of the treatment [3]. The evidence to support the use of iontophoresis to treat pain and inflammation associated with musculoskeletal injuries remains limited [4–8]. However, most research that investigates the efficacy of iontophoresis focuses on the parameters of drug delivery rather than examining the current parameters to optimize treatment. Factors such as skin permeability and passive diffusion based on the concentration of the medication should be considered as well as the electro-repulsive components to determine the best method of clinical application of iontophoresis [1,9]. Although *in vitro* studies are effective in determining the ability of electrical current to enhance the diffusion across synthetic membranes [10], they do not consider the time that the medication is in contact with the skin that may affect the overall treatment outcome.

Using Coulomb's Law, the dosage for the electrical component of iontophoresis is described in milliamp minutes (mA*min) or the amplitude of the galvanic current (milliamps or mA) multiplied by amount of time delivered (minutes). Treatment guidelines suggested by manufacturers imply that both factors of the dosage (amplitude and time) will result in similar affects on drug delivery, although this hypothesis has not been tested clinically. Most commercial units electronically adjust the treatment time after the clinician adjusts the current amplitude based on the perceived comfort by the patient. The amplitudes available in these devices are generally well tolerated so patients often request a shorter treatment time with higher amplitude current to minimize the time needed in the clinic. The iontophoresis parameters vary widely in clinical trials, ultimately affecting the ability to standardize clinical treatments to comprehensively evaluate the effectiveness of this modality. Therefore, it is important to mechanistically determine which component of the dosage, either time or current amplitude, has a greater affect on drug absorption.

The purpose of this study was to compare the reduction in cutaneous sensation following two iontophoresis protocols with the same 40 mA*min dosage to a sham treatment. Both iontophoresis treatments used 2% lidocaine HCL and were delivered with a high amplitude/short duration (HASD) or a low amplitude/long duration (LALD) method. The amount of skin anesthesia following each treatment was measured using Semmes-Weinstein monofilaments (SWM).

## 2. Experimental Section

A double-blind crossover design was used for this study. Twenty-one healthy subjects (13 male, 8 female); (age 21.2 ± 4.3 years; height 170.7 ± 10.3 cm, mass 82.1 ± 19.2 kg) volunteered for this study. Subjects had no known allergies to lidocaine or adhesives, there were no neurological pathology of the upper body and participants were free from skin abnormalities in the area of electrode placement. The Institutional Review Board for Health Sciences Research approved the study and all subjects signed informed consent forms prior to enrollment.

The independent variables were the treatment condition: (1) HASD: 40 mA*min with 2% lidocaine HCL applied at an amplitude of 4 mA for 10 min; (2) LALD: 40 mA*min with 2% lidocaine HCL at an amplitude of 2 mA for 20 min; (3) Sham: zero amplitude for 20 min using 2% lidocaine HCL. All conditions utilized standard commercial iontophoresis electrodes with 2 mL of 2% lidocaine HCL injected into the bladder of the active, positive electrode. The HASD treatment was delayed for 10 min prior to application to make the testing time consistent among groups. Before each condition and within 5 min following each condition, SWM were used to quantify cutaneous sensation by an investigator who was blinded to the treatment condition. Each treatment was separated by 48 hours and we alternated the application to the right and left forearm at each condition to prevent a cumulative effect between treatments.

The order of treatments was randomly assigned and the order was counterbalanced. The dependent variable was score on the SWM test (Smith and Nephew, Inc., Germantown, WI), which has been validated for use in sensory research [11,12]. The monofilaments were applied to the skin with enough force to cause each filament to buckle into the shape of a crescent moon. SWM exam started with the smallest diameter monofilament, testing every other diameter monofilament until the monofilament was perceived. When a “yes” response was achieved, the next smallest monofilament was tested. If that monofilament received a “yes” response then that diameter was recorded. If the next smallest monofilament received a “no” response then the monofilament that received a “yes” response was recorded. A no-touch condition during testing was randomly incorporated. This procedure has a reported intertester and intratester reliability of 92% [13] and 89% [14] respectively with a sensitivity of 70% and specificity of 90% [15]. There are no units for the SWM since the value is associated with the logarithm of the force produced, expressed in tenths of a milligram [16]. Neither the subject nor the clinician administering the test was aware of the condition assigned, and the participant was draped during the sensory exam so that he or she could not see the test being performed.

The Phoresor II Auto Model PM850 (IOMED, Salt Lake City, UT) was used to deliver the direct current using medium-size TransQE electrodes (IOMED, Salt Lake City, UT). The areas of electrode placement were cleaned with an alcohol pad and dried. The active electrode with the medication applied was placed on the volar aspect of the anterior forearm, 4 inches distal to the anti-cubital crease. The dispersive, negative electrode was placed on the same arm, 4 inches proximal to the anti-cubital crease. A mark was made to indicate the bladder portion of the active electrode. All monofilament testing was done within that demarcation to provide consistency.

A two-way ANOVA with repeated measures was used to examine the effects of amplitude and duration on the SWM score. The independent variables were the condition (HASD, LALD, and sham) and test (pre and post). The dependent variable was smallest monofilament diameter perceived in the treatment area. The a priori alpha level was set at P < 0.05. Post Hoc pairwise comparisons were performed to explain significant interactions.

## 3. Results and Discussion

**Figure 1.**
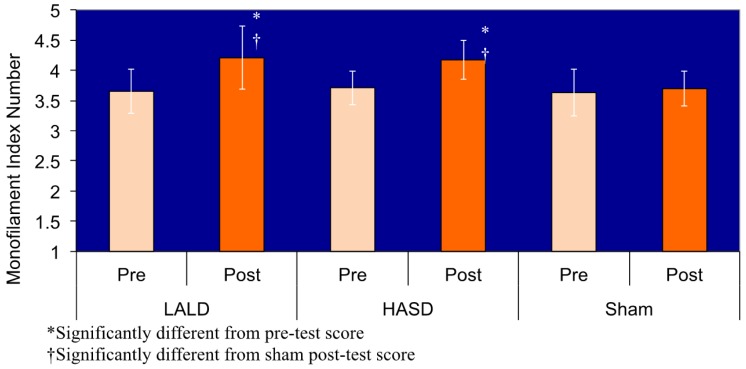
Means of Semmes Weinstein monofilament scores by condition and time.

**Figure 2.**
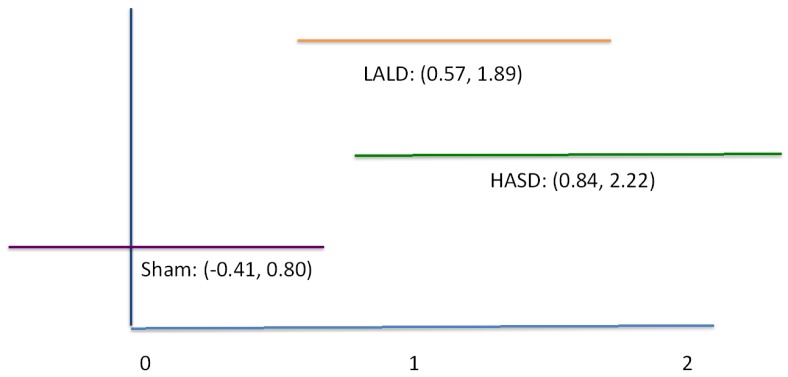
Confidence Intervals of each condition, indicating strong effects of both the high amplitude/short duration (HASD) and low amplitude/long duration (LALD) conditions.

Data are presented in Table 1. There was a significant test by condition interaction (F_2,40_ = 6.950, P = 0.003). Pairwise comparisons revealed that there were no significant differences among any of the pretest measures, nor was there a difference in the pre and posttest scores in the sham condition. There was a significant difference between the post-test sensation measures for the LALD compared to the control condition (P = 0.001), as well as a significant difference between the HASD and control condition (P = 0.001), graphically represented in Figure 1. Both the HASD and the LALD conditions had strong effect sizes. However there was no significant difference between LALD and HASD post-test scores. Confidence intervals are reported in Figure 2.

Although many pharmaceutical agents can be used with iontophoresis, we chose to use lidocaine so that the effects of manipulating the electrical current parameters could be examined in a non-invasive manner. Lidocaine or other anesthetics have been used in this fashion to examine the effects of either phonophoresis [17] or iontophoresis [18–20] mechanistically. Since the dosage or concentration of the drug, the site of application, and the total amount of charge applied for each condition remained constant, we were able to determine the effects of manipulating the current parameters on the overall iontophoretic effect. Similar to previous investigations, we estimated the drug absorption to be associated with the degree of cutaneous anesthesia in this model [18]. Our results showed that both the HASD and the LALD conditions resulted in significantly greater anesthesia than both pre-tests and the sham condition, but there was no superiority of either method. Thus, as hypothesized based on Coulomb's Law, the total charge applied affected the results, rather than the manipulation of the either the magnitude of the current or the duration of the stimulation.

The rate-limiting factor for any transdermal drug delivery system is the stratum corneum of the skin. This outermost layer is comprised of keratinized cells and has both lipophilic and hydrophilic properties to reduce fluid loss and prevent the absorption of most topical agents [21]. Several strategies have been developed to improve transdermal drug delivery and include methods to change the barrier properties of the stratum corneum [1], to improve the hydration of the skin [22], or to provide a phyical enhancement techniques such as employed by iontophoresis, phonophoresis or electroporation [23]. Iontophoresis requires the pharmaceutical agent to be in an ionic form and utilizes an electrorepulsion mechanism of low amplitude galvanic current to drive the desired medication through the skin. The medication in its ionic form must be the same polarity as the active electrode. The primary mechanisms of enhanced transport is through existing pathways such as the hair follicles and sweat glands [24] and is often dependent on the amount or concentration of the drug in its ionic form [25]. Phonophoresis uses ultrasound energy to enhance the transport of whole molecules through the skin [22,26] while electroporation uses a high voltage current of short duration to allow enhanced diffusion of topical agents [27]. Both phonophoresis and electroporation are hypothesized to temporarily change the structure of the stratum corneum to enhance the penetration of the drug.

Factors that are proposed to affect iontophoresis include the physiochemical properties of the pharmacological agent such as the concentration, molecular charge and the molecular size.[28] Other factors, such as characteristics of the skin should also be considered, particularly if there is a potential osmotic effect, similar to electroporation following the application of current. For example, in electroporation, there are changes noted in the structure of the stratum corneum [23] and these changes have been observed in conjunction with increased permeability of the skin with various current waveforms, including biphasic [10,29,30]. From a clinical perspective, it would stand to reason that if the electrical current acts on the skin, and the longer the medication is in contact with the skin, the greater the chance of transport through the skin using a combination of the mechanisms presented with iontophoresis and electroporation [27,31,32].

This mechanism is being addressed by new commercially produced clinical units, although there is little data in the literature to determine their best use for musculoskeletal pathologies. With this treatment, current is applied via a “patch” electrode containing the pharmaceutical agent, and the medication remains in place for several hours after the treatment, exploiting the passive absorption potential. This application would more closely mimic the LALD condition, and has been shown to be a factor for continued absorption of lidocaine after the current had ceased [33]. Conversely, the HASD condition might permit a greater absorption of the drug as a result of a greater electrorepulsion factor of the higher electrical stimulation apmplitude [1]. We did not see a difference in the amount of drug absorption when the medication remained in contact with the skin for a longer time. Thus, using this model, the electroporetic effect on the skin was likely to minimal, and we observed an iontophoretic effect based on the total charge.

The dosages that we chose to investigate were similar to clinical applications in the treatment of musculoskeletal pathologies. The variation in the length of time between the doses was 10 min. Although increased passive transport may have occurred within this timeframe, it may have been too short of a difference between the two conditions to be able to assess a measureable difference. Likewise, we did not investigate the duration of the anesthesia that may further indicate passive diffusion into the dermis. Future studies should broaden the variation in the application time or test the difference between a standard iontophoresis treatment and an electrophoretic treatment. The electrophoretic treatment would incorporate a short electrical stimulation time and much longer topical drug appliation (for hours). Lidocaine would be an unlikely surrogate to represent drug absorption in that type of study since the half-life of lidocaine is approximately 90 min [34]. Furthermore, lidocaine with epinepherine should be explored since the epinepherine would prevent pooling of the drug in the capillaries.

Iontophoresis has been shown to be an effective method of lidocaine delivery for decreasing cutaneous sensation [18,35,36]. Lidocaine blocks the fast-gated sodium channels to inhibit presynaptic neurons from depolarizing [34]. Thus, there is an elimination of all sensory information, including pain when the drug is absorbed into the dermis. The solution of lidocaine HCL was not designed for topical administration, which would likely require some agent such as a chemical enhancer to improve the transport through the stratum corneum. The diminished cutaneous response, as measured by the SWM indicated increased absorption when the iontophoresis conditions were used, compared to the sham, which had no change in anesthesia. Since the sensory effects were diminished, rather than eliminated, we anticipated that we would be able to evaluate a difference in the two treatment conditions using the SWM scores. In other words, we did not achieve a ceiling effect with the measurement tool.

There is generally no consensus in the literature on the manipulation of the amplitude of the current or the duration of the treatment [37]. The clinician determines the overall dosage, typically 40 mA*min, and the current amplitude is adjusted to the patient's comfort level. There is a maximum amplitude on commercial devices to reduce the risk of adverse effects. Often, patients choose a shorter iontophoresis treatment (with a higher amplitude) to reduce the overall time required. Chemical burns and heat burns have been reported using clinical parameters of iontophoresis [38], however, we did not observe any cutaneous changes such as redness at the electrode sites in either condition. Since there was no difference in the overall treatment effect, clinicians should continue to use patient feedback, particularly for those with sensitive skin to determine their current amplitude. None of our participants reported any discomfort from the HASD treatment.

## 4. Conclusions

Using this model of HASD and LALD, there does not appear to be an effect of manipulating the individual components of the iontophoresis parameters when the same dosage is applied. However, we did not examine the length of time that anesthesia would last. Our results imply that the clinician can increase the amplitude of stimulation within patient tolerance to minimize the treatment time, or choose a more comfortable, longer duration to elicit an effective iontophoresis application.

## Figures and Tables

**Table 1.** Means and standard deviations by condition.

**Condition**	**Test**	**SWM Mean ± SD**	**Effect Size (Cohen's*d*)**
Sham	Pre	3.63 ± 0.39	0.20
	Post	3.70 ± 0.29	
HASD	Pre	3.71 ± 0.28	1.52
	Post	4.17 ± 0.32*	
LALD	Pre	3.65 ± 0.37	1.24
	Post	4.21 ± 0.52*	
